# A predictive model for depression in Chinese middle-aged and elderly people with physical disabilities

**DOI:** 10.1186/s12888-024-05766-4

**Published:** 2024-04-23

**Authors:** Lianwei Shen, Xiaoqian Xu, Shouwei Yue, Sen Yin

**Affiliations:** 1 Rehabitation Center, Qilu Hospital of Shandong University, 250000 Jinan, Shandong China; 2 Neurology Department, Qilu Hospital of Shandong University, Jinan, China

**Keywords:** Depression, Physical disabilities, Clinical prediction models, CHARLS

## Abstract

**Background:**

Middle-aged and older adults with physical disabilities exhibit more common and severe depressive symptoms than those without physical disabilities. Such symptoms can greatly affect the physical and mental health and life expectancy of middle-aged and older persons with disabilities.

**Method:**

This study selected 2015 and 2018 data from the China Longitudinal Study of Health and Retirement. After analyzing the effect of age on depression, we used whether middle-aged and older adults with physical disabilities were depressed as the dependent variable and included a total of 24 predictor variables, including demographic factors, health behaviors, physical functioning and socialization, as independent variables. The data were randomly divided into training and validation sets on a 7:3 basis. LASSO regression analysis combined with binary logistic regression analysis was performed in the training set to screen the predictor variables of the model. Construct models in the training set and perform model evaluation, model visualization and internal validation. Perform external validation of the model in the validation set.

**Result:**

A total of 1052 middle-aged and elderly persons with physical disabilities were included in this study, and the prevalence of depression in the elderly group > middle-aged group. Restricted triple spline indicated that age had different effects on depression in the middle-aged and elderly groups. LASSO regression analysis combined with binary logistic regression screened out Gender, Location of Residential Address, Shortsightedness, Hearing, Any possible helper in the future, Alcoholic in the Past Year, Difficulty with Using the Toilet, Difficulty with Preparing Hot Meals, and Unable to work due to disability constructed the Chinese Depression Prediction Model for Middle-aged and Older People with Physical Disabilities. The nomogram shows that living in a rural area, lack of assistance, difficulties with activities of daily living, alcohol abuse, visual and hearing impairments, unemployment and being female are risk factors for depression in middle-aged and older persons with physical disabilities. The area under the ROC curve for the model, internal validation and external validation were all greater than 0.70, the mean absolute error was less than 0.02, and the recall and precision were both greater than 0.65, indicating that the model performs well in terms of discriminability, accuracy and generalisation. The DCA curve and net gain curve of the model indicate that the model has high gain in predicting depression.

**Conclusion:**

In this study, we showed that being female, living in rural areas, having poor vision and/or hearing, lack of assistance from others, drinking alcohol, having difficulty using the restroom and preparing food, and being unable to work due to a disability were risk factors for depression among middle-aged and older adults with physical disabilities. We developed a depression prediction model to assess the likelihood of depression in Chinese middle-aged and older adults with physical disabilities based on the above risk factors, so that early identification, intervention, and treatment can be provided to middle-aged and older adults with physical disabilities who are at high risk of developing depression.

## Introduction

With the development of global economy, many countries, including China, are facing serious aging problems [1]. Physical disabilities seem to be inevitable in middle-aged and elderly people as they age. A survey study showed that the overall disability rate of activities of daily living (ADLs) among middle-aged and elderly people in China was 23.8%, and the overall disability rate of organic ADLs was 35.4% [2]. And depression is very common among the middle-aged and elderly population, which is the most important psychological problem of middle-aged and elderly people [3]. In China, the prevalence of depression in the middle-aged and elderly population over 45 years old is more than 30% [4]. By 2023, depression will rank first in the global burden of disease [5], bringing a serious economic burden to patients, families, and society. At the same time, large-scale population-based research studies have shown that middle-aged and older adults with disabilities, especially women, have higher rates of depression than non-disabled older adults [6–7]. The Peruvian National Health Survey showed that middle-aged and older persons with disabilities tend to suffer from more severe depression [8]. This suggests that depression is prevalent and severe in the middle-aged and older physically disabled population. However, most studies on depression in people with disabilities suffer from the problem of including too few correlates or failing to quantify the risk of correlates in depression [9–10]. Also, relevant studies are unable to screen potentially at-risk patients for depression and fail to achieve prevention of depression in people with disabilities. We screened national survey data from the China Longitudinal Study of Health and Retirement (CHARLS) for correlates of previous studies that may affect depression among people with disabilities. Afterwards, we constructed a predictive model of depression among middle-aged and elderly physically disabled people in China by combining LASSO regression and binary logistic regression to screen out predictor variables that were highly correlated with depression. After verifying the credibility, stability, and generalizability of the model, we visualized the model as a nomogram, and the nomogram obtained from the model visualization can help middle-aged and elderly people with disabilities to self-check whether they have high risk factors for depression and intervene on their own. Based on the nomogram of the prediction model, the clinical staff can quickly screen out the middle-aged and elderly persons with physical disabilities who have high risk of depression, so as to achieve early identification, early intervention and early treatment of depression.

## Method

### Data sources and model design

**Fig. 1:**
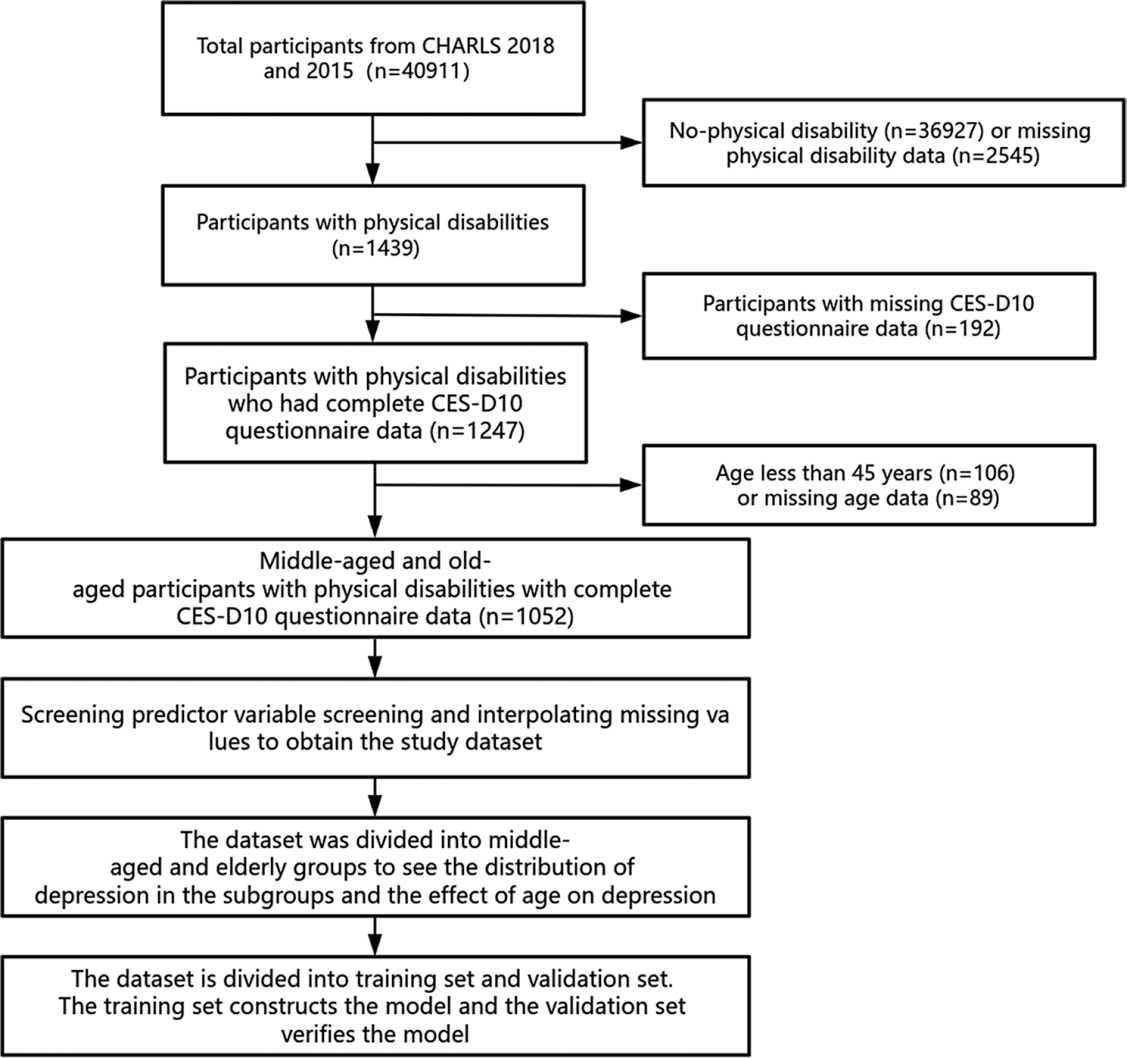
Flowchart

CHARLS is a large-scale interdisciplinary survey project hosted by the National Development Research Institute of Peking University, and jointly implemented by the China Social Science Survey Center of Peking University and the Peking University Youth League Committee. Its purpose is to collect a nationally representative set of longitudinal survey data representing households and individuals of middle-aged and elderly people aged 45 and above in China, which can be used to analyze the population aging problem in China, promote interdisciplinary research on aging, and provide a more scientific basis for China to formulate and improve relevant policies [11]. In this study, we selected data from 2015 to 2018, from which we extracted data on the Center for Epidemiologic Studies Depression Scale (CES-D10), health behaviors, demographic factors, physical functioning, and social interactions of middle-aged and older adults with physical disabilities. The data screening process is shown in Fig. 1 Flowchart.

### Depression assessment

The CES-D10 contains 10 items, each scored: 0 (rarely or not at all), 1 (sometimes), 2 (most of the time), 3 (all of the time). The total score ranges from 0 to 30, with lower scores indicating lower levels of depressive symptoms. Studies have shown that a threshold of 10 has reasonable sensitivity and specificity for Chinese older adults [12]. Therefore, we defined a CES-D10 score ≥ 10 as depression [13].

### Definition of middle-aged and older persons and definition of physical disability

Middle age was defined as 45 to 65 years old, and older people were defined as older than 65 years old [14–18]. Physical disability was defined as loss of function or dysfunction of the human locomotor system to varying degrees due to disability of the limbs or paralysis or deformity of the trunk of the limbs [19].

### Correlation between age and depression

We used Association Between Age and depression Using a Restricted Cubic Spline Regression Model after looking at the prevalence of depression in the middle-aged and older age groups in our data set. Graphs show ORs for depression according to Age. Data were fitted by a logistic regression model, and the model was conducted with 3 knots at the 10th, 50th, 90th percentiles of Age.

### Inclusion of predictor variables and data set creation

Combining previous studies and the principle of no more than 30% missing values for predictor variables. For demographic factors, we chose gender, childhood health and residential address. Gender differences in depression have been demonstrated in many studies. However, a global meta-analysis showed that gender differences in depression are evident in adolescence [20]. Therefore, we also included the childhood health status of the sample.A multi-country systematic evaluation showed that the association between urban and rural residence and depression remained significant after adjusting for covariates [21]. For health behaviors, we chose sleep duration, nap time, smoking, alcohol consumption, and weekly exercise. Population suffering from depression is associated with dysregulation of normal sleep-wake mechanisms [22]. Systematic evaluations have shown that smoking and alcohol consumption increase the risk of depression [23–24]. Exercise, on the other hand, reduces the risk of depression [25]. For physical functioning, we chose vision, hearing, inability to work due to disability, in dressing, bathing, eating, getting up, using the toilet, controlling urination and defecation, preparing food, shopping, and completing household chores. The Spanish National Health Survey showed that visual impairment and hearing impairment lead to more severe depression [26]. Decreased ability to perform ADLs in the population and dependence on ADLs have also been shown to be associated with depression [27]. In terms of socialization, we choose whether or not to get help in the future. Good social relationships can play a role in preventing depression, especially in the elderly [28]. After the collection of predictor variables for the dataset was completed, multiple interpolation of missing values was performed.

### General characteristics of the data set

We used chi-square tests for dichotomous predictor variables in the dataset, independent samples t-tests for measured variables, and Mann-Whitney U-tests for ordered categorical variables.

### LASSO regression analysis combined with binary logistic regression for analytical screening of predictor variables

We randomly divided the dataset into training and validation sets in the ratio of 7:3. In the training set, we performed LASSO regression analysis using the R package “glmnet” (version 4.2.2). After an initial screening of the variables in the training set, binary logistic regression analyses were performed on the above variables using SPSS (version 26).

### Model construction

After screening the predictor variables for the predictive model of physical disability depression in middle-aged and elderly Chinese, we constructed the model in Rstudio using the lrm function in the design package. And we visualized the model using the “nomogram” function in the “rms” package.

### Comprehensive evaluation of the model

#### Model evaluation

In binary classification problems, we often use the Concordance Index (C-Index) to measure the model’s ability to correctly rank positive and negative examples. The C-Index can take values between 0.5 and 1, where 0.5 indicates random prediction and 1 indicates perfect prediction. A higher C-index implies that the model has better ranking performance and discrimination [29]. In addition, a calibration curve is a graphical tool used to assess the calibration performance of a prediction model. It helps researchers to understand the level of predictive accuracy of a model under different probability intervals by comparing the relationship between actual observations and model predictions. We calculated the C_index of the model in order to assess the discrimination of the model; calibration curves of the model were produced to assess the calibration of the model. However, it is difficult to determine the ideal value of the C-index to ensure the efficacy of the predictive model, and it is equally difficult to determine what erroneous calibration values should be rejected. Therefore, we chose Decision Curve Analysis (DCA) to further evaluate the model. It is a graphical tool for evaluating the decision-making performance of a classification model under different thresholds, which can help us in weighing the predictions of the classification model and determining the most suitable thresholds for decision-making [30]. Also, in order to comprehensively assess the performance of the model in different aspects (coverage, accuracy), we also calculated the Recall, Precision, F1_score, and Brier scores.

#### Internal validation

Internal validation is a necessary step in the development of a model, the significance of which is to quantify the predictive performance of the developed model. In this study, internal validation is carried out using Bootstrap resampling method. Bootstrap resampling is a statistical method used to estimate the distribution of sample statistics as well as parameter uncertainty by reusing the data set by means of random sampling from the training set with put-backs. This method is effective in assessing the stability and reliability of a model over different subsets of data and providing confidence intervals for parameter estimates [31]. We set the sample size to 1000 to obtain more accurate estimates.

#### External validation

The model is externally validated using the C-index and calibration curves in the validation set.

## Results

### General characteristics of the data set

A total of 1052 middle-aged and older adults with physical disabilities were included in this study, of whom 497 did not suffer from depression (47.2%) and 555 suffered from depression (52.8%). The distribution of predictor variables is shown in Table 1.

**Table 1:** General characteristics of the data set

Predictor variable	Depression (*n* = 555)	Non-depressed (*n* = 497)	Pearson squared/Z	*P*
Gender n (%)				
Male (0) Female (1)	283(50.99) 272(49.01)	313(62.98) 184(37.02)	15.341^a^	<0.001
Location of Residential Address n (%)				
Urban (0) Rural (1)	91(16.40) 464(83.60)	111(22.33) 386(77.67)	5.958^a^	0.015
Farsightedness n (%)				
good functionality(0) poor functionality(1)	355(63.96) 200(36.04)	410(82.49) 87(17.51)	45.386^a^	<0.001
Shortsightedness n (%)				
good functionality(0) poor functionality(1)	363(65.41) 192(34.59)	411(82.70) 86(17.30)	40.319^a^	<0.001
Hearing n (%)				
good functionality(0) poor functionality(1)	411(74.05) 144(25.95)	448(90.14) 49(9.86)	45.295^a^	<0.001
Evaluate Health during Childhood n (%)				
NO(0) YES(1)	505(90.99) 50(9.01)	478(96.38) 19(5.62)	11.506^a^	0.001
Intensive Physical Activity More than 10 min Each Week n (%)				
NO(0) YES(1)	451(81.26) 104(18.74)	408(82.09) 89(17.91)	0.121^a^	0.728
Moderate Physical Activity More than 10 min Each Week n (%)				
NO(0) YES(1)	397(71.53) 158(28.47)	377(75.86) 120(24.14)	2.521^a^	0.112
Light Physical Activity More than 10 min Each Week n (%)				
NO(0) YES(1)	123(22.16) 432(77.84)	59(11.87) 438(88.13)	19.408^a^	<0.001
Any possible helper in the future n (%)				
NO(0) YES(1)	218(39.28) 337(60.72)	130(26.16) 367(73.84)	20.396^a^	<0.001
Smoked n (%)				
NO(0) YES(1)	467(84.14) 88(15.86)	425(85.51) 72(14.49)	0.381^a^	0.537
Hours of sleep per day (x ± s)	5.85 ± 0.11	6.37 ± 0.08	-0.3.79	<0.001
Hours of daily nap (x ± s)	0.67 ± 0.03	0.54 ± 0.03	2.43	0.015
Alcoholic in the Past Year n (%)				
never or very rarely(1) less than 1 time per month(2) more than 1 per month(3)	258(46.49) 53(9.54) 244(43.97)	315 (63.38) 43 (8.65) 139 (27.97)	-5.688	<0.001
Difficulty with Dressing n (%)				
No difficulties(1) Difficult, can still be done(2) Difficult, need help(3) Unable to complete(4)	393(70.81) 99(17.84) 41(7.39) 22(3.96)	403 (81.09) 43 (8.65) 30 (6.04) 21 (4.22)	-3.492	<0.001
Difficulty with Bathing or Showering n (%)				
No difficulties(1) Difficult, can still be done(2) Difficult, need help(3) Unable to complete(4)	367(66.12) 64(11.53) 86(15.50) 38(6.85)	382 (76.86) 35 (7.04) 45 (9.05) 35 (7.05)	-3.508	<0.001
Difficulty with Eating n (%)				
No difficulties(1) Difficult, can still be done(2) Difficult, need help(3) Unable to complete(4)	486(87.57) 51(9.19) 12(2.16) 6(1.08)	450 (90.54) 20 (4.02) 14 (2.82) 13 (2.62)	-1.331	0.183
Difficulty with Getting into or out of Bed n (%)				
No difficulties(1) Difficult, can still be done(2) Difficult, need help(3) Unable to complete(4)	399(71.89) 113 (20.36) 31 (5.59) 12 (2.16)	407 (81.89) 47 (9.46) 16 (3.22) 27 (5.43)	-3.313	0.001
Difficulty with Using the Toilet n (%)				
No difficulties(1) Difficult, can still be done(2) Difficult, need help(3) Unable to complete(4)	313 (56.4) 164(29.55) 45 (8.11) 33 (5.94)	350 (70.42) 82 (16.5) 28 (5.63) 37 (7.45)	-3.990	<0.001
Difficulty with Controlling Urination and Defecation n (%)				
No difficulties(1) Difficult, can still be done(2) Difficult, need help(3) Unable to complete(4)	461(83.06) 57 (10.27) 18 (3.24) 19 (3.43)	450 (90.54) 21 (4.23) 8 (1.61) 18 (3.62)	-3.393	0.001
Difficulty with Household Chores n (%)				
No difficulties(1) Difficult, can still be done(2) Difficult, need help(3) Unable to complete(4)	277(49.91) 86 (15.5) 59 (10.63) 133(23.98)	338 (68.01) 35 (7.04) 29 (5.84) 95 (19.11)	-5.064	<0.001
Difficulty with Preparing Hot Meals n (%)				
No difficulties(1) Difficult, can still be done(2) Difficult, need help(3) Unable to complete(4)	339(61.08) 58 (10.45) 36 (6.49) 122(21.98)	348 (70.02) 30 (6.04) 18 (3.62) 101 (20.32)	-2.537	0.011
Difficulty with Shopping for Groceries n (%)				
No difficulties(1) Difficult, can still be done(2) Difficult, need help(3) Unable to complete(4)	361(65.05) 43 (7.75) 37 (6.67) 114 (20.53)	388 (78.07) 16 (3.22) 16 (3.22) 77 (15.49)	-4.256	<0.001
Unable to work due to disability n (%)				
YES(1) NO(2) Too old for work(3)	268(48.29) 202(36.39) 85 (15.32)	130 (26.16) 267 (53.72) 100 (20.12)	-6.447	<0.001

*Note* Variables with suffixes “0” and “1” are dichotomous variable.Variables with suffixes “1–4” are ordered categorical variables. *P* < 0.001 means that when the statistical analysis yields a very small p-value, shown as 0.000, it is expressed in the table as *P* < 0.001

### Correlation between age and depression

**Fig. 2:**
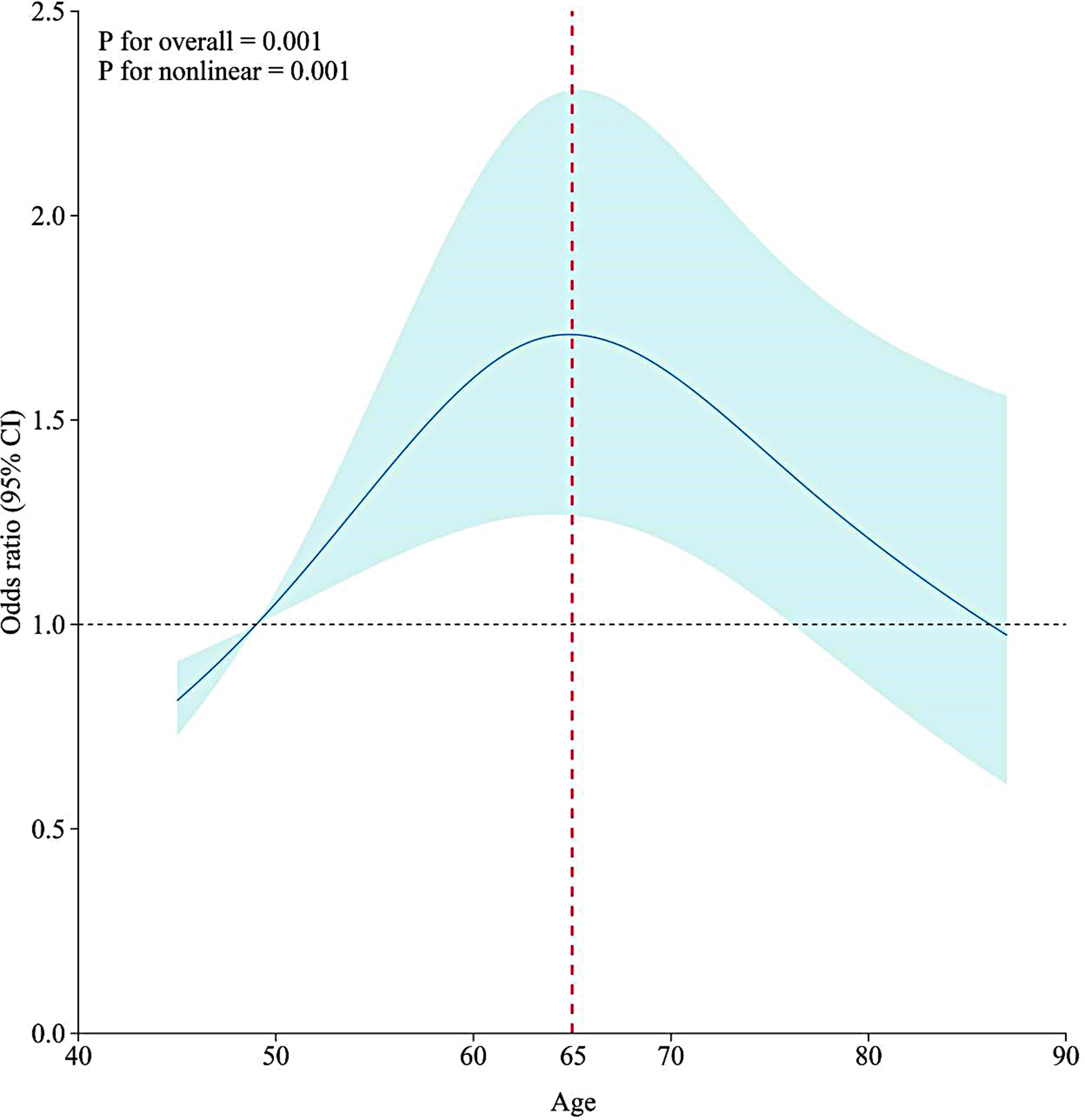
Restricted Cubic Spline Regression Model *Note* Solid lines indicate ORs, and shadow shape indicate 95% CIs. *OR* odds ratio; *CI* confidence interval

In the middle-aged group, there were 648 people, 335 (51.6%) suffered from depression. The older age group had a total of 404 individuals and 220 (54.5%) suffered from depression. We plotted the Restricted Cubic Spline Regression Model between age and depression (Fig. 2). For the middle-aged group, age between 45 and 50 years was a protective factor and greater than 50 years was a risk factor, and the correlation was stronger with increasing age. For the older age group, the correlation was weaker with age.

### LASSO regression analysis combined with binary logistic regression analysis to screen predictor variables

**Fig. 3:**
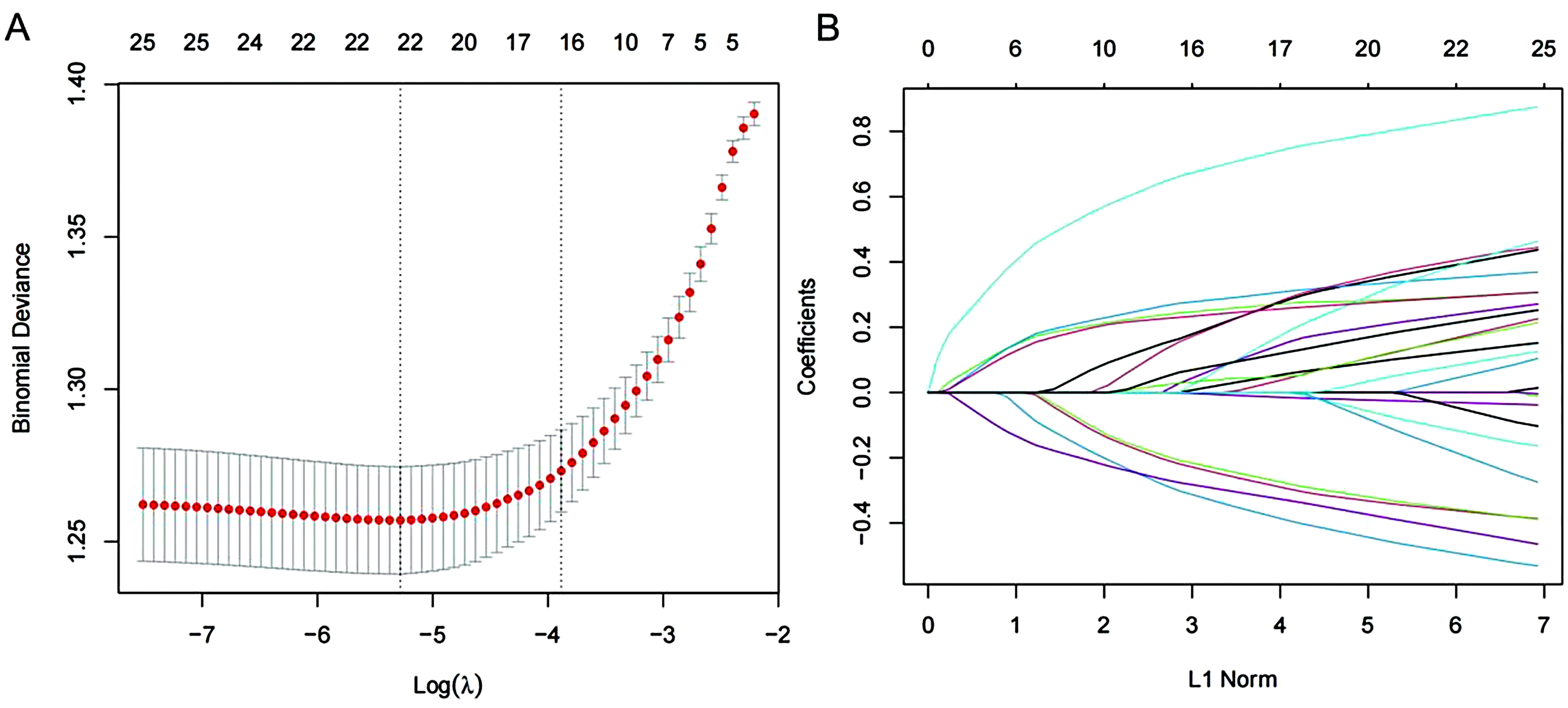
LASSO regression analysis combined with binary logistic regression for analytical screening of predictor variables (**A**) LASSO Selection Path Plot: Vertical dashed lines on the left side of the plot indicate Log(λ) corresponding to the minimum error (lambda.1se), while vertical dashed lines on the right side of the plot indicate Log(λ) that is one standard error away from the minimum error (lambda.min). Binomial Deviance: binomial distribution loss function of the model computed on each fold during the cross-validation process (**B**) LASSO Path Plot: the curve of regression coefficients versus Log(λ) as the coefficient scores are gradually decreasing. Coefficients: regression coefficients corresponding to each independent variable.L1 Norm: the use of the absolute value to Calculate the number of norms for all eigenfactors of the model

In the LASSO selection path diagram (Fig. 3A), two specific values of λ, lambda.min and lambda.1se, are shown.The LASSO path diagram (Fig. 3B) shows that as the coefficients decrease, the predictors decrease accordingly. We screened 22 predictor variables based on lambda.1se in Fig. 3A. Eighteen statistically significant variables were screened by SPSS, and the covariance diagnostic scores were all less than 10, indicating that the 18 variables were independent. The 18 predictor variables were then included in the binary logistic regression analysis, in which the ordered categorical variables were set as covariates, and the results are shown in Table 2. The significance of the Hosmer-Lemeshaw test was 0.819, indicating a good model fit. According to the principle of *P* < 0.10 [32], we screened out Gender, Location of Residential Address, Shortsightedness, Hearing, Any possible helper in the future, Alcoholic in the Past Year, Difficulty with Using the Toilet, Difficulty with Preparing Hot Meals, and Unable to work due to disability totaled 9 variables from Table 2 to construct the model.

**Table 2:** Results of binary logistic regression analysis

Predictive variables	B	P	OR(95%CI)
Hours of sleep per day	-0.040	0.303	0.961 ( 0.890, 1.037 )
Gender	0.319	0.079	1.376 ( 0.964, 1.964 )
Location of Residential Address	-0.402	0.074	0.669 ( 0.431, 1.039 )
Farsightedness	0.166	0.455	1.180 ( 0.764, 1.823 )
Shortsightedness	0.387	0.077	1.473 ( 0.959, 2.262 )
Hearing	0.831	0.001	2.296 ( 1.398, 3.771 )
Evaluate Health during Childhood	0.376	0.276	1.457 ( 0.741, 2.865 )
Light Physical Activity More than 10 min Each Week	-0.342	0.171	0.710 ( 0.435, 1.160 )
Any possible helper in the future	-0.437	0.017	0.646 ( 0.451, 0.925)
Alcoholic in the Past Year		0.006	
Alcoholic in the Past Year(1)	-0.510	0.009	0.601 (0.410, 0.880)
Alcoholic in the Past Year(2)	-0.188	0.550	1.207 (0.651, 2.238)
Difficulty with Bathing or Showering		0.566	
Difficulty with Bathing or Showering(1)	-0.692	0.203	0.500 (0.172, 1.454)
Difficulty with Bathing or Showering(2)	-0.656	0.262	0.519 (0.165, 1.634)
Difficulty with Bathing or Showering(3)	-0.344	0.514	0.709 (0.252, 1.993)
Difficulty with Eating		0.601	
Difficulty with Eating(1)	0.849	0.334	2.337 (0.418, 13.082)
Difficulty with Eating(2)	1.192	0.200	3.295 (0.531, 20.424)
Difficulty with Eating(3)	1.070	0.326	2.915 (0.344, 24.711)
Difficulty with Using the Toilet		0.099	
Difficulty with Using the Toilet(1)	0.983	0.058	2.674 (0.967, 7.393)
Difficulty with Using the Toilet(2)	1.260	0.017	0.905 (1.252, 9.937)
Difficulty with Using the Toilet(3)	0.754	0.184	0.388 (0.699, 6.462)
Difficulty with Controlling Urination and Defecation		0.356	
Difficulty with Controlling Urination and Defecation(1)	-0.606	0.300	0.545 (0.173, 1.715)
Difficulty with Controlling Urination and Defecation(2)	-0.199	0.769	0.820 (0.218, 3.082)
Difficulty with Controlling Urination and Defecation(3)	-0.124	0.882	1.132 (0.220, 5.842)
Difficulty with Household Chores		0.321	
Difficulty with Household Chores(1)	-0.413	0.292	0.662 (0.307, 1.426)
Difficulty with Household Chores(2)	-0.145	0.734	1.156 (0.501, 2.670)
Difficulty with Household Chores(3)	-0.149	0.750	0.862 (0.344, 2.155)
Difficulty with Preparing Hot Meals		0.098	
Difficulty with Preparing Hot Meals(1)	0.972	0.015	2.643 (1.207, 5.787)
Difficulty with Preparing Hot Meals(2)	0.619	0.180	1.858 (0.752, 4.593)
Difficulty with Preparing Hot Meals(3)	0.814	0.128	2.258 (0.792, 6.441)
Difficulty with Shopping for Groceries		0.680	
Difficulty with Shopping for Groceries(1)	-0.174	0.615	0.840 (0.426, 1.656)
Difficulty with Shopping for Groceries(2)	0.027	0.957	1.027 (0.391, 2.695)
Difficulty with Shopping for Groceries(3)	0.379	0.444	1.461 (0.553, 3.860)
Unable to work due to disability		0.001	
Unable to work due to disability(1)	0.894	0.002	2.444 (1.407, 4.244)
Unable to work due to disability(2)	0.206	0.472	1.229 (0.700, 2.157)

### Constructing and validating a predictive model of depression for middle-aged and elderly persons with physical disabilities in China

**Fig. 4:**
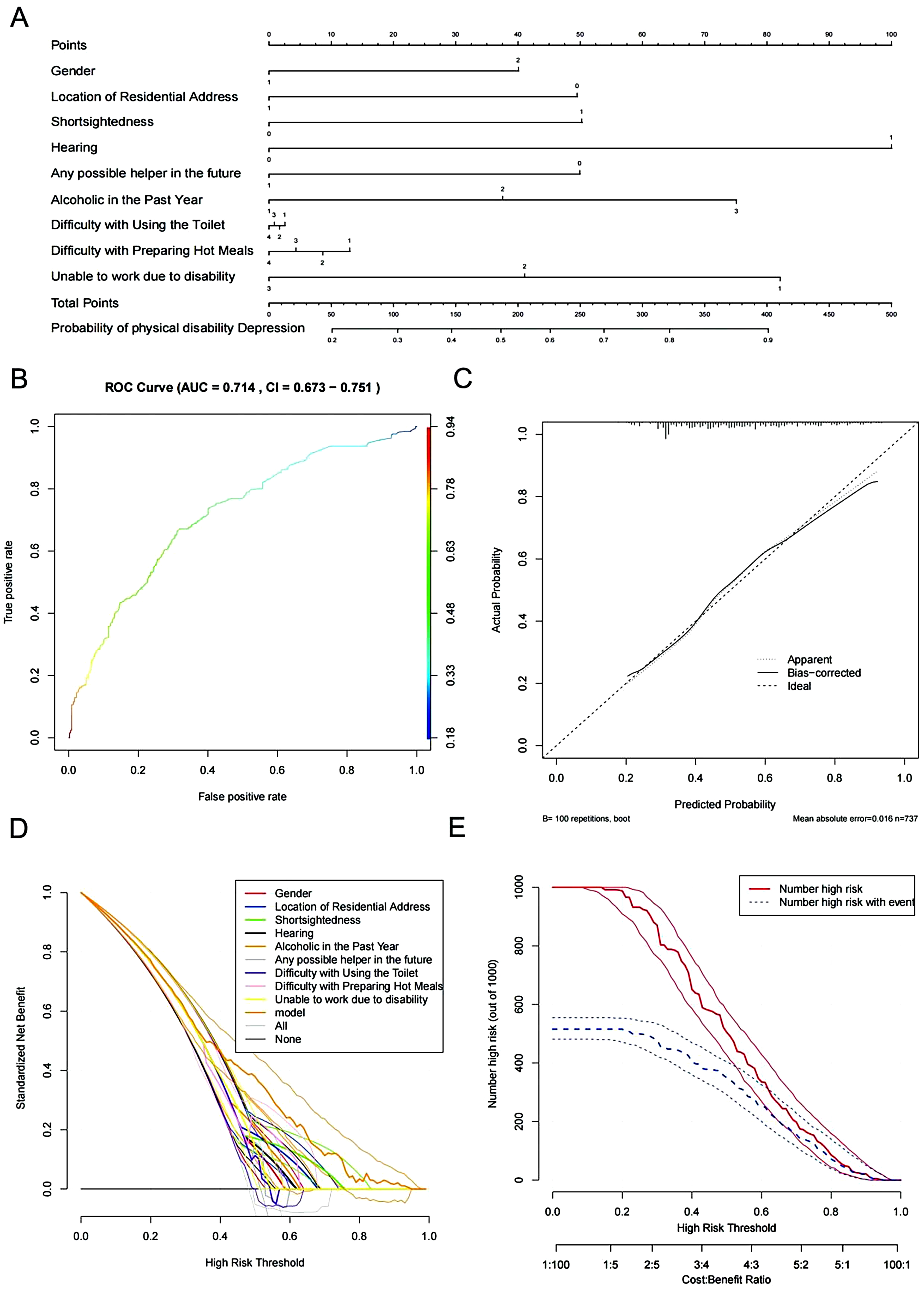
Construction and validation of the depression prediction model for middle-aged and elderly physically disabled people in China (**A**) nomogram: the sum of the scores on each predictor, which predicts the probability that depression will occur (**B**) ROC plot of the model: the horizontal coordinate is the false positive rate, representing the proportion of false positive samples. The vertical coordinate is the sensitivity, representing the proportion of true positive samples. The two form the ROC curve, which is used to assess the model’s ability to correctly classify positive and negative samples at different classification thresholds (**C**) Calibration curves: the horizontal coordinate is the probability of an event occurring as predicted by the model. The vertical coordinate is the proportion of events that actually occur within the predicted probability range. The calibration curve is used to assess the agreement between the predicted probability of an event and the actual probability of its occurrence (**D**) DCA chart: the horizontal coordinate is the high risk threshold, referring to the thresholds selected for the different predictor variables. The vertical coordinate is the standardised net benefit, which refers to the standardised net benefit calculated for the different predictor variables constituting the model and the line strategy. The DCA plot of the two makes it possible to assess the extent to which each model outperforms or underperforms the baseline strategy under different decision scenarios (**E**) Net Benefit Curve: The high-risk thresholds and cost-benefit ratios in the horizontal coordinate compare the costs of using the model with its benefits. The vertical coordinate represents the number of samples judged to be depressed at the selected high-risk threshold for a sample size of 1,000. The three form a net benefit curve that can be used to assess the benefits of each model across different predictor variables, helping policymakers to make optimal decisions

After determining the final variables for constructing the depression prediction model for Chinese middle-aged and elderly persons with physical disabilities, we constructed the model in Rstudio using the “rms” package and generated a nomogram using the “nomogram” function of the package (Fig. 4A). The area under the ROC curve of the depression prediction model for Chinese middle-aged and elderly persons with physical disabilities was 0.714 (95% CI: 0.673–0.751), as shown in Fig. 4B. The Recall of the model was 0.655, the Precision was 0.692, the F1_score was 0.672, and the Brier score was 0.213. The mean of the mean calibration curve was absolute error was 0.016, as shown in Fig. 4C. The DCA curves showed that, over a wide range of thresholds, the model constructed using the model containing nine predictor variables had higher vertical coordinate values than the model constructed using a single predictor variable. This suggests that the predictive model constructed in this study has a greater gain, see Fig. 4D. The net gain curve shows that patients using the predictor variables have a higher risk of depression, see Fig. 4E.

### Internal validation of the depression prediction model for middle-aged and elderly people with physical disabilities in China

**Fig. 5:**
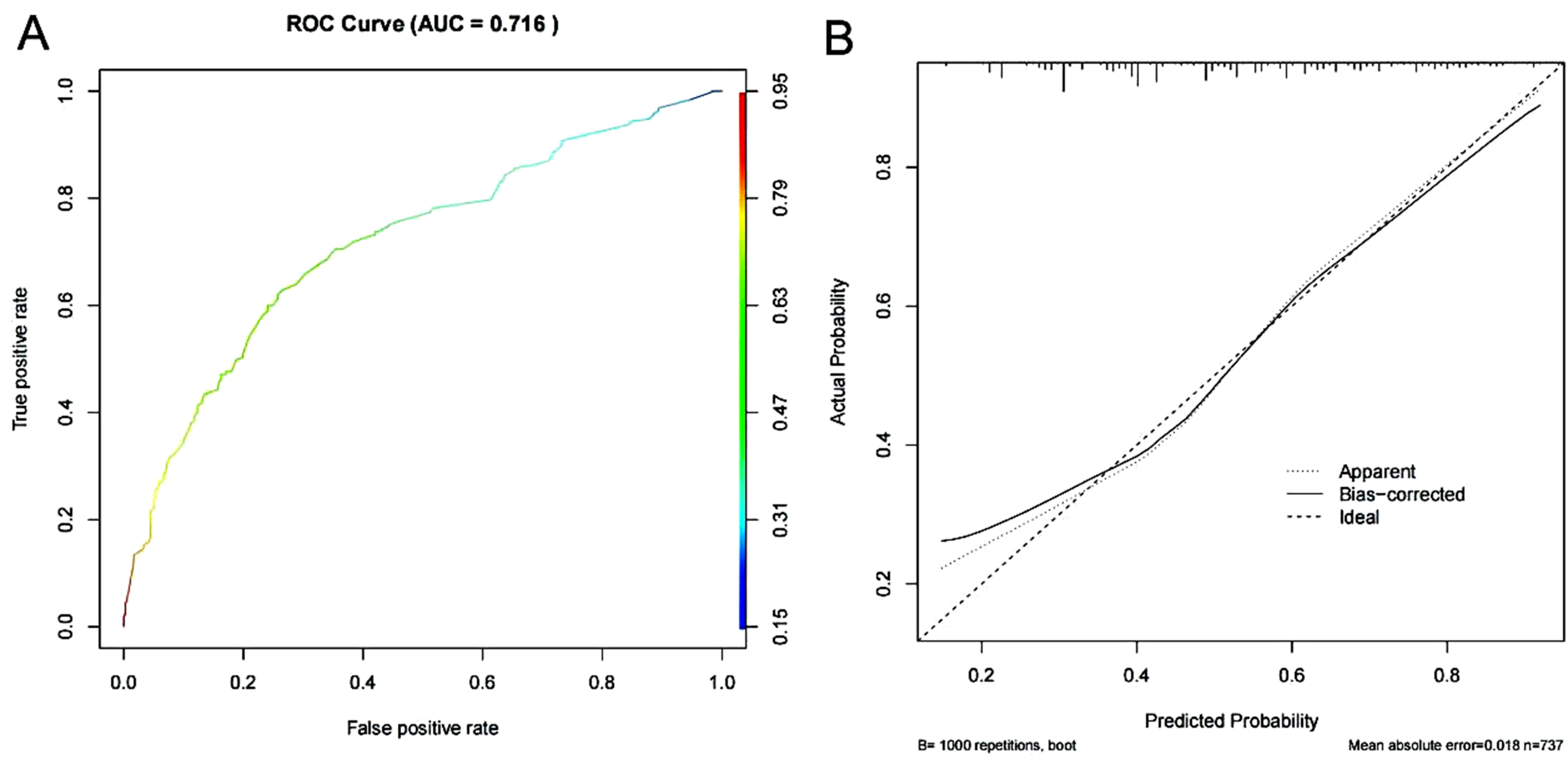
Internal validation of the depression prediction model for middle-aged and elderly Chinese people with physical disabilities (**A**) ROC plot of internal validation, see Fig. 4B for picture annotations (**B**) Calibration curves of internal validation, participate in Fig. 4C for picture annotations

The area under the ROC curve for internal validation was 0.716, see Fig. 5A. The mean absolute error of the calibration curve was 0.018, see Fig. 5B.

### External validation of the depression prediction model for middle-aged and elderly people with physical disabilities in China

**Fig. 6:**
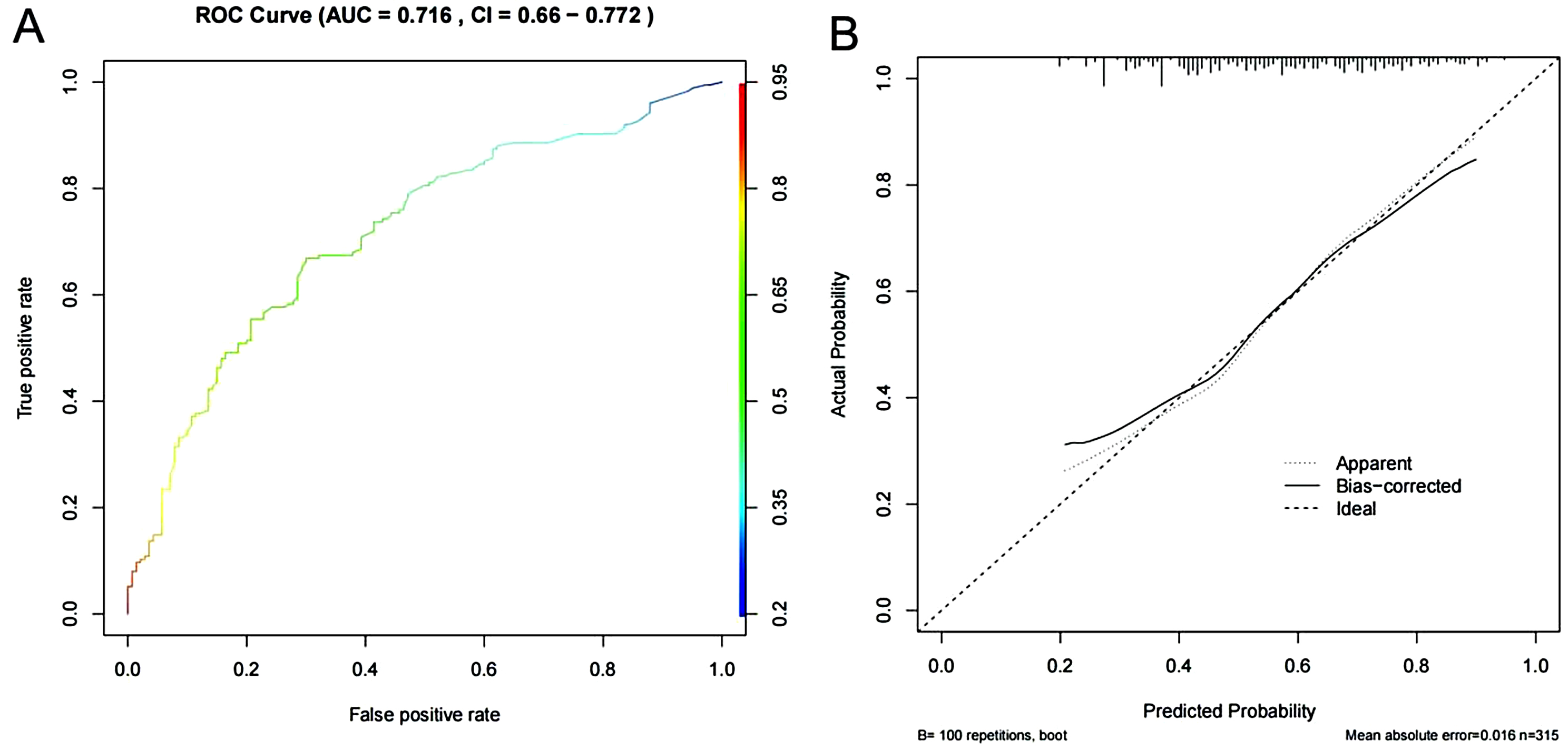
External validation of the depression prediction model of Chinese middle-aged and elderly persons with physical disabilities (**A**) ROC plots of the depression prediction model of Chinese middle-aged and elderly persons with physical disabilities in the validation set, see Fig. 4B for the picture annotations (**B**) Calibration curves of the depression prediction model of Chinese middle-aged and elderly persons with physical disabilities in the validation set, see Fig. 4C for the picture annotations

We externally validated the model according to its predictor variables in the validation set. In the validation set, the area under the ROC curve of the depression prediction model for Chinese middle-aged and elderly persons with physical disabilities was 0.716 (95% CI: 0.660–0.772), as shown in Fig. 6A. The mean absolute error of the calibration curve was 0.016, as shown in Fig. 6B.

## Discussion

Researchers in the CHARLS database used a multi-stage sampling method with probability proportional to size to select 150 county-level units sequentially from all counties in China, and ultimately to the community level. This sampling method ensures the representativeness and reliability of the sample and allows for effective statistical analysis of the entire Chinese population [11]. Table 1 shows that the prevalence of depression among middle-aged and elderly persons with disabilities in China is more than 50 per cent, and more than 80 per cent of the patients live in rural areas where there is a lack of medical protection. The prevalence of depression is higher in the elderly group than in the middle-aged group. For the middle-aged group, age between 45 and 50 years is a protective factor, and age over 50 years is a risk factor, and the risk increases with age. For the elderly group, age was a risk factor, but the risk decreased with increasing age. In summary, depression is a serious problem for middle-aged and elderly Chinese people with disabilities.

In the training set, LASSO regression analyses were combined with binary logistic regression analyses to screen for nine variables that were highly correlated with depression.The area under the ROC curve for the model, internal validation and external validation were all greater than 0.70, the mean absolute error was less than 0.02, and the recall and precision were both greater than 0.65, indicating that the model performs well in terms of discriminability, accuracy and generalisation. The DCA curve and net gain curve of the model indicate that the model has high gain in predicting depression. F1_score was 0.672, which indicated that the model could take into account both Recall and Precision. indicates that the model is able to balance Recall and Precision. Brier score of 0.213 indicates that the model is more accurate in predicting the probability of occurrence of depression.

The nomogram shows that being female, living in a rural area, having poor vision and/or hearing, lack of help from others, drinking alcohol, having difficulty in using the bathroom and preparing food, and being unable to work due to a disability are risk factors for depression among middle-aged and older adults with physical disabilities. Women have long been recognized as an independent risk factor for depression. Epidemiology shows that the prevalence of depression in women is almost twice as high as in men [33]. The causes of this phenomenon include genetically determined vulnerability, hormonal fluctuations associated with various aspects of reproductive function, and hypersensitivity to hormonal fluctuations that mediate depressive states [33]. Data from the Korean Longitudinal Survey on Aging showed that the female physical disability group exhibited more depressive symptoms than the male physical disability group [34]. However, this did not occur in the non-disabled group [34]. The higher prevalence of depression and the greater severity of symptoms in rural older adults compared to urban older adults may be due to under-recognition and inadequate treatment [35]. Factors affecting income play an important role in the development of depression in rural residents [36]. It is clear that middle-aged and older adults with physical disabilities will have lower incomes than middle-aged and older adults without physical disabilities. The Spanish National Health Survey showed that the prevalence and severity of depression in adults with both visual and hearing impairments were higher than those with either impairment alone [26]. Nomogram suggests that hearing impairment and visual impairment provide a very large contribution to elevating the incidence of depression in middle-aged and older adults with disabilities. Social relationships not only influence the onset of depression, but also have a significant impact on the severity of depression [28]. Good social relationships can play a protective role against the onset of depressive symptoms in old age [28]. However, middle-aged and elderly patients with physical disabilities are more likely to lack good social relationships than middle-aged and elderly patients without physical disabilities. This is one of the reasons for the high prevalence of depression in the middle-aged and elderly population with physical disabilities. Chronic intake of excessive alcohol may affect the neurological function of the brain and metabolic changes, increasing the prevalence and severity of depression [24]. Epidemiologic studies have shown that physically disabled people have higher alcohol intake [37]. Chronic intake of excessive alcohol may affect the neurological function of the brain and metabolic changes, increasing the prevalence and severity of depression [24]. Combined with the nomogram, alcohol consumption plays an important role in depression among middle-aged and elderly Chinese with physical disabilities. Difficulties in accessing the toilet and preparing food represent the dependence of physically disabled people on the most basic activities of daily living. The interaction between depression and ADLs is unclear in the general population [27]. However, in the disabled population, disability-induced declines in ADLs can exacerbate depression [27]. Finally, the inability to work due to disability directly affects income, which plays an important role in the onset of depression [36].

From the predictor variables, it can be seen that female middle-aged and elderly people with disabilities living in rural areas are at high risk of depression and need to be paid attention by the society. Meanwhile, we constructed a credible predictive model of depression among Chinese middle-aged and elderly persons with disabilities, and its Nomogram can be used as an efficient screening tool for depression among middle-aged and elderly persons with disabilities.

Our study also has limitations. On the one hand, there are a large number of missing values in the CHARLS database regarding the presence of physical disability, which prevented us from calculating the prevalence of physical disability. On the other hand, our model was developed based on data from China, making it difficult to generalize this study to other countries.

## Conclusion

This study showed that being female, living in rural areas, having poor vision and/or hearing, lack of assistance from others, drinking alcohol, having difficulty using the restroom and preparing food, and not being able to work due to a disability were risk factors for depression among middle-aged and older adults with physical disabilities. In summary, we screened predictor variables that were highly associated with depression among middle-aged and elderly Chinese physical disabilities people and constructed a prediction model. Based on the above risk factors, we can assess the probability of depression among middle-aged and elderly physically disabled people. Middle-aged and elderly people can reduce the risk of depression by intervening in the risk factors themselves. Clinical staff can quickly identify individuals with physical disabilities at high risk of depression based on the nomogram of the prediction model, thus enabling early identification, early intervention, and early treatment of depression. Also, our data were obtained from a nationally representative data set, and the findings emphasize the urgent need for depression prevention and treatment for female middle-aged and elderly physical disabilities people living in rural areas.

## Data Availability

The data for this article comes from the the China Health and Retirement Longitudinal Study database for 2018. Available from https://charls.pku.edu.cn/en/.

## Abbreviations

ADLs: activities of daily living.
CHARLS: The China Health and Retirement Longitudinal Study.
DCA: Decision Curve Analysis.
CES-D10: Center for Epidemiologic Studies Depression Scale.
C-Index: Concordance Index.
